# Emerging roles of miRNAs in neuropathic pain: From new findings to novel mechanisms

**DOI:** 10.3389/fnmol.2023.1110975

**Published:** 2023-02-17

**Authors:** Yu-Ying Zhao, Zi-Jun Wu, Li-Juan Zhu, Tong-Xiang Niu, Bin Liu, Jing Li

**Affiliations:** ^1^Department of Anesthesiology, Tianjin Medical University General Hospital, Tianjin, China; ^2^Tianjin Research Institute of Anesthesiology, Tianjin, China; ^3^Department of Critical Care Medicine, General Hospital of Tianjin Medical University, Tianjin, China; ^4^Center for Critical Care Medicine, Institute of Hematology and Blood Diseases Hospital, Chinese Academy of Medical Science and Peking Union Medical College, Tianjin, China

**Keywords:** neuropathic pain, miRNAs, exosomes, neuroinflammation, review

## Abstract

Neuropathic pain, which results from damage to the somatosensory nervous system, is a global clinical condition that affects many people. Neuropathic pain imposes significant economic and public health burdens and is often difficult to manage because the underlying mechanisms remain unclear. However, mounting evidence indicates a role for neurogenic inflammation and neuroinflammation in pain pattern development. There is increasing evidence that the activation of neurogenic inflammation and neuroinflammation in the nervous system contribute to neuropathic pain. Altered miRNA expression profiles might be involved in the pathogenesis of both inflammatory and neuropathic pain by regulating neuroinflammation, nerve regeneration, and abnormal ion channel expression. However, the lack of knowledge about miRNA target genes prevents a full understanding of the biological functions of miRNAs. At the same time, an extensive study on exosomal miRNA, a newly discovered role, has advanced our understanding of the pathophysiology of neuropathic pain in recent years. This section provides a comprehensive overview of the current understanding of miRNA research and discusses the potential mechanisms of miRNAs in neuropathic pain.

## Introduction

Neuropathic pain (NP), which results from damage to the somatosensory nervous system, is a global clinical condition that affects many people worldwide (Scholz et al., 2019). Neuropathic pain is associated with significant economic and public health burdens and is often difficult to manage because the underlying mechanisms remain obscure. However, mounting evidence indicates a role for neurogenic inflammation and neuroinflammation in pain patterns development (Yi et al., 2021). There is increasing evidence that the activation of neurogenic inflammation and neuroinflammation both in the periphery and the central nervous system conduce to the maintenance of neuropathic pain (Walters, 2014; Huh et al., 2017). Our earlier research looked at whether chronic inflammation brought on by an inflammatory reaction in the neurological system brought on by nerve damage might be the source of chronic neuropathic pain (Li et al., 2013).

Recent evidence has suggested that the anti-inflammatory effects of microRNAs are essential for pathogenesis in various contexts, such as inflammation, tumours, respiratory and cardiovascular diseases (Bernardo et al., 2015; Rupaimoole and Slack, 2017; Li Y. et al., 2019; Hill and Tran, 2021). Unfortunately, the pathogenesis of neuropathic pain has not been fully elucidated. Using microarray techniques, recent studies have reported that miRNAs play pivotal roles in the evolution and progression of neuropathic pain by regulating neuronal excitability and plasticity, neuronal ion channels, neuroinflammation, synaptic plasticity and other functions. These results indicate that altered miRNA expression profiles might be involved in the pathogenesis of neuropathic pain. However, the lack of knowledge about miRNA target genes prevents the achievement of a full understanding of the biological functions of miRNAs under these conditions. This review provides insight into miRNA expression profiling studies on neuropathic pain. We also highlight the molecular mechanisms of specific miRNAs that play critical functional roles in neuropathic pain pathogenesis and discuss their potential diagnostic, prognostic, and therapeutic utilization in neuropathic pain clinical management.

## The structure and function of miRNAs

### Gene structure

**Figure 1:**
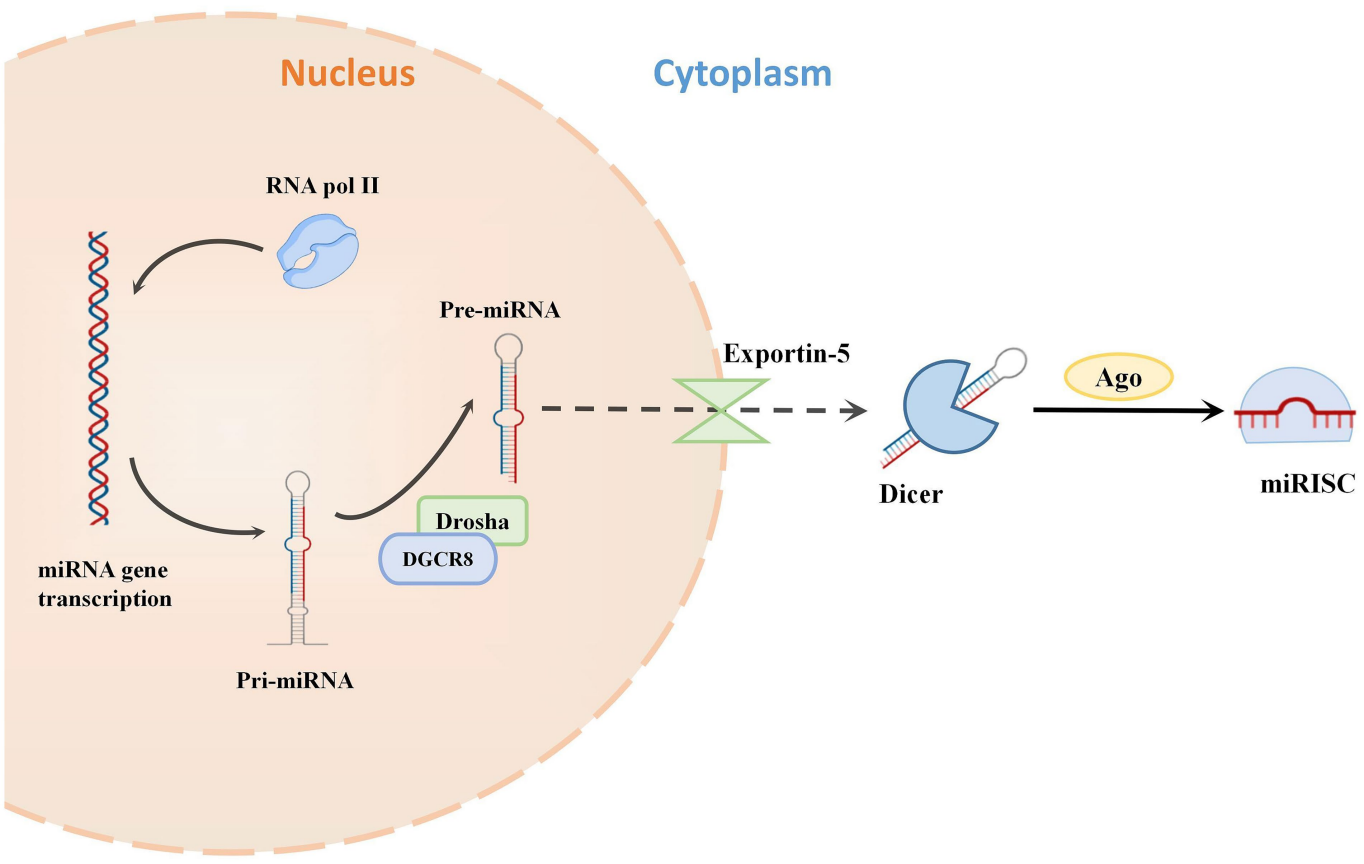
The classical bioformation process of miRNA. miRNAs are typically transcribed as primary miRNAs (pri-miRNAs) by RNA polymerase II (Pol II). The microprocessor complex, composed of the RNase III enzyme Drosha and the double-stranded RNA (dsRNA)-binding protein (dsRBP) DiGeorge critical region 8 (DGCR8), cleaves the pri-miRNA and releases a precursor miRNA (pre-miRNA). The export receptor exportin 5 binds pre-miRNAs and aids their export into the cytoplasm. Then the RNase III endonuclease DICER cleaves the pre-miRNA to release double-stranded miRNA. This miRNA is cleaved by Argonaute (Ago) to form the miRNA-induced silencing complex (miRISC).

MicroRNAs are a varied family of 19–24 nucleotide short noncoding single-stranded RNAs that have fundamental biological activities in posttranscriptional gene silencing. The ribonuclease II enzymes, Drosha and Dicer sequentially cleave precursor RNA transcripts to generate miRNAs (Hutvágner et al., 2001; Gregory et al., 2004; Han et al., 2004). The miRNA-induced silencing complex (miRISC) is then formed by loading miRNAs onto the effector protein Argonaute (Ago) (Schwarz et al., 2003). By using translational repression and/or mRNA instability, miRISC silences mRNA targets by binding to them *via* sequence complementarity (Schwarz et al., 2003; Macfarlane and Murphy, 2010; Figure 1).

### The function of miRNAs

MiRNAs, which regulate approximately one-third of human genes, are widely present in mammalian cells. More than 1800 miRNAs, which target 60% of human mRNAs, are found in the human genome (Olive et al., 2015). Numerous human disorders, including neuropathic pain, are linked to mutations in miRNA genes and miRNA pathway genes. Furthermore, the peripheral and central nervous systems, which contain pain-related components, are widely dispersed with microRNAs. Despite not being directly engaged in peptide synthesis, miRNAs are a type of noncoding RNAs that have a significant impact on miRNA stability and protein translation (Saliminejad et al., 2019).

By binding to the 3’UTR of target mRNAs, miRNAs control posttranscriptional gene expression by lowering the majority (84%) of protein products (Lutz et al., 2014). One miRNA can target more than one mRNA transcript, and multiple miRNAs can simultaneously act on one mRNA strand (Lutz et al., 2014; Li et al., 2015). There is a general consensus that miRNAs play pivotal roles in regulating important biological processes, including early development, cell proliferation, cell death and apoptosis, fat metabolism, cell differentiation, and disease progression (Lima et al., 2011). Various disorders, including cardiovascular, cerebrovascular, and neurodegenerative diseases, may be impacted by changed miRNA expression profiles, according to the results of experimental models (Barwari et al., 2016; Wang et al., 2020).

## Potential regulatory mechanisms of miRNAs in neuropathic pain

**Figure 2:**
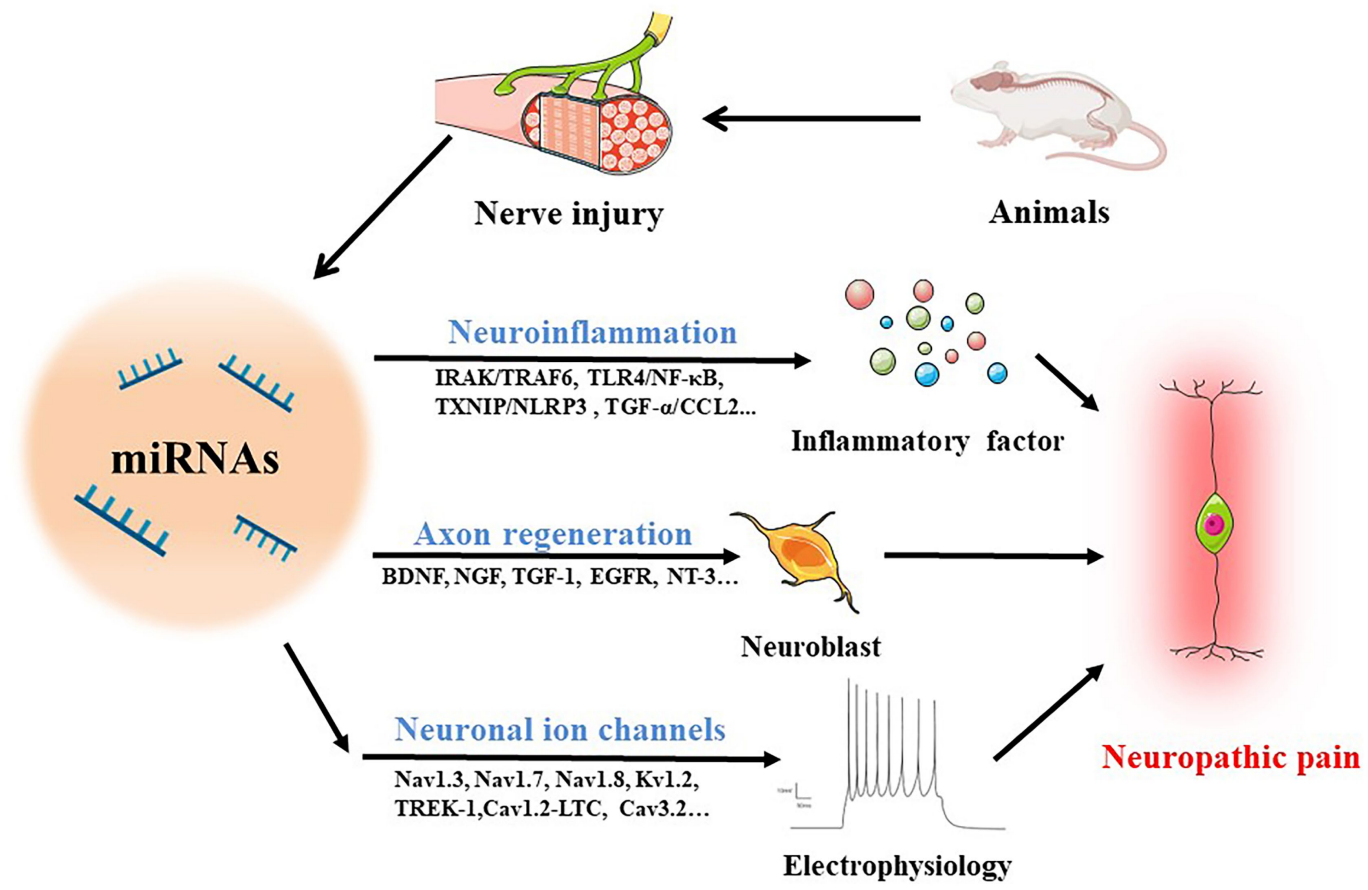
MiRNAs in neuropathic pain. The following routes are used by miRNAs to mediate neuropathic pain in animals with damaged nerves: (1) operating on immune cells to stimulate the production of inflammatory cytokines through IRAK/TRAF6, TLR4/NF-κB, TXNIP/NLRP3, TGF-α/CCL2 and other pathways to regulate neuroinflammation; (2) accelerating axon regeneration by increasing the synthesis of trophic factors such BDNF, NGF, TGF-1, EGFR, and NT-3; (3) mediating neuroelectrophysiological changes by activating ion channels like Nav1.3, Nav1.7, Nav1.8, Kv1.2, TREK-1, Cav1.2-LTC, and Cav3.2.

Several studies have reported broad abnormalities in miRNAs in animals following peripheral nerve injury. Several identified miRNAs are involved in neuroinflammation, nerve regeneration, and abnormal ion channel expression (Figure 2), suggesting that deregulation of miRNA expression may be included in the development of neuropathic pain and could be potentially useful diagnostic markers, improving the classification of neuropathic pain. The absence of information on miRNA target genes, however, limits a complete comprehension of the biological roles played by miRNAs. This section gives a thorough overview of what is currently known about miRNAs and explores how they can contribute to neuropathic pain (Table 1).

**Table 1:** miRNAs and neuropathic pain.

Mechanisms	miRNA	Target	Objectives	Models	Results	References
*Neuroinflammation*						
Infiltration of immune cells	miR-590-3p ↑	RAP1A ↓	C57BL/6 mice	DPNP	T cell infiltration was reduced, which in turn hinders DPNP progression.	Wu et al. (2020)
	miR-214-3p ↑	CSF1 ↓	SD rats	SNL	Attenuated the neuroinflammation and pain behavior.	Liu L. et al. (2020)
	miR-146a-5p ↑	IRAK1 / TRAF6 ↓	SD rats	CCI	Inhibited the development of CCI-induced neuropathic pain.	Wang et al. (2018)
Neuroinflammation	miR-381 ↑	HMGB1 and CXCR4 ↓	SD rats	CCI	Inhibited neuropathic pain development.	Zhan et al. (2018)
	miR-362-3p ↑	BAMBI ↓	C57BL/6 mice	CCI	NP progression was suppressed.	Zhang et al. (2022)
	miR-183 ↑	TGF-α/CCL2/CCR2 ↓	C57BL/6 mice	OA	Osteoarthrotic pain was ameliorated.	Tao et al. (2021)
	miR-28-5p ↑	ZEB1 ↓	SD rats	CCI	Reduced neuropathic pain.	Bao et al. (2018)
	miR-128-3p ↑	ZEB1 ↓	SD rats	CCI	Alleviated the progression of neuropathic pain	Zhang et al. (2020)
	miR-136 ↑	ZEB1 ↓	SD rats	CCI	Inhibited neuropathic pain development.	Shen et al. (2019)
	miR-150 ↑	ZEB1 ↓	SD rats	CCI	Inhibited neuropathic pain *in vivo*.	Yan et al. (2018a,b)
	miR-200b/	ZEB1 ↓	SD rats	CCI	Reduced neuropathic pain development *in vivo*.	Yan et al. (2018a,b)
	miR-429 ↑					
	miR-23a ↑	CXCR4/TXNIP/NLRP3 ↓	C57BL/6 mice	pSNL	Inhibited neuropathic pain development.	Pan et al. (2018)
	miR-140 ↑	S1PR1 ↓	SD rats	CCI	Suppressed CCI-stimulated neuropathic pain.	Li et al. (2021)
	miR-144 ↑	RASA1 ↓	C57BL/6 mice	CCI	Facilitated the inhibition of neuropathic pain development.	Zhang et al. (2020)
	miR-216a-5p ↑	KDM3A ↓	SD rats	CCI	Alleviated neuropathic pain in rats.	Wang and Li W. et al. (2021)
	miR-93 ↑	STAT3 ↓	SD rats	CCI	Inhibited neuropathic pain development of CCI rats.	Yan et al. (2017)
	miR-155 ↓	SOCS1 ↑	SD rats	CCI	Attenuated neuropathic pain.	Tan et al. (2015)
	miR-155 ↓	TRPA1 ↓	SD rats	Chemotherapeutic Oxaliplatin	Supprressed the OXL-induced neuropathic pain.	Miao et al. (2019)
	miR-221 ↓	SOCS1 ↑	SD rats	CCI	Alleviateed neuropathic pain and neuroinflammation.	Xia et al. (2016)
	miR-221 ↓	SOCS3 ↑	SD rats	DPN	Reduced pain and decreased expression of inflammatory factors.	Wu et al. (2021)
	miR-22 ↑	Mtf1 ↑	Kunming mice	CFA	Promoted the development and maintenance of inflammatory pain.	Hao et al. (2022)
*Nerve regeneration*	miR-192-5p ↑	XIAP ↑	SD rats	SNI	Decreased the apoptosis of nerve cells, and promote the repair and regeneration of peripheral nerve injury.	Liu X. et al. (2020)
	miR-210 ↑	EFNA3 ↓	*CF*-1 mice	SNC	Promoted sensory axon regeneration and inhibit apoptsosis.	Hu et al. (2016)
	miR-135s ↑	KLF4 ↓	C57BL/6 mice	ONI	Facilitated RGC axon regeneration after optic nerve injury in adult mice.	van Battum et al. (2018)
	miR-125b ↑	JAK/STAT ↓	mice	SCI	Promoted the repair and regeneration following spinal cord injury.	Dai et al. (2018)
	miR-155 ↓	SPRR1A ↑	C57BL/6 mice	SCI	Enhanced neuron survival and axon growth.	Gaudet et al. (2016)
	miR-19a ↑	PTEN ↑	SD rats and C57BL/6 mice	ONC	Promoted axon regeneration after optic nerve crush in adult mice.	Mak et al. (2020)
	miR-21 ↑	PTEN ↓	SD rats	SNI	Promoted neurite growth.	Kar et al. (2021)
	miR-21 ↑	TGFβI/TIMP3/EPHA4 ↓	SD rats	Nerve injury	Promoted SC proliferation and axon regeneration.	Ning et al. (2020)
	miR-21 ↑	EGFR ↑	SD rats	ONC	Enhanced the axon regeneration after ONC.	Li et al. (2018)
	miR-199a-3p ↑	mTOR ↓	SD rats	SNI	Attenuated neurite growth.	Kar et al. (2021)
	miR-26a ↑	GSK3β/Smad1 ↓	*CF*-1 mice	SNC	Supported mammalian axon regeneration *in vivo*.	Jiang et al. (2015)
	miR-455-5p ↓	GSK3β/Tau ↑	Rats	SNI	Promoted axonal growth and regeneration.	Su et al. (2020)
	miR let-7 ↑	Ntn1 ↓	SD rats	SNI	Reduced axon outgrowth.	Wang et al. (2019)
	miR-9 ↑	Dcc ↓				
	miR-9 ↑	FoxP1 ↓	*CF*-1 mice	SNC	Inhibited axon regeneration *in vitro* and *in vivo*.	Jiang et al. (2017)
	miR-138 ↓	SIRT1 ↑	*CF*-1 mice	SNC	Promoted mammalian axon regeneration.	Liu et al. (2013)
*Neuronal ion channels*						
Voltage-gated sodium channel	miR-7a ↑	β2 subunit ↓	SD rats	SNL and CCI	Suppressed neuropathic pain.	Sakai et al. (2013)
	miR-96 ↑	Nav1.3 ↓	SD rats	CCI	Alleviated neuropathic pain.	Chen et al. (2014)
	miR-384-5p ↑	SCN3A ↓	SD rats	CCI	Significantly repressed mechanical allodynia and heat hyperalgesia in CCI rats.	Ye et al. (2020)
	miR-182 ↑	Nav1.7 ↓	SD rats	SNI	Alleviated SNI-induced neuropathic pain.	Cai et al. (2018)
	miR-30b ↑	Nav1.3 ↓	SD rats	SNL	Suppressed neuropathic pain.	Su et al. (2017)
	miR-30b-5p ↑	Nav1.6 ↓	SD rats	Chemotherapeutic Oxaliplatin	Attenuated pain.	Li L. et al. (2019)
	miR-30b ↑	Nav1.7 ↓	SD rats	SNI	Alleviated neuropathic pain.	Shao et al. (2016)
	miR-183 ↑	Nav1.3/Nav1.7/Nav1.8 ↓	C57BL/6 mice	DMM	Inhibited the expression of pain-related factors and ameliorated OA pain.	Tao et al. (2021)
Voltage-gated potassium channels	miR-17-92 ↓	multiple voltage-gated potassium channels ↑	SD rats	SNL	Alleviated mechanical allodynia induced by nerve injury.	Sakai et al. (2013)
	miR-137 ↓	Kv1.2 ↑	SD rats	CCI	Alleviated mechanical allodynia and thermal hyperalgesia.	Zhang et al. (2021)
	miR-183-5P ↑	TREK-1 ↓	SD rats	CCI	Efficiently ameliorated neuropathic pain.	Shi et al. (2018)
Voltage-gated calcium channels	miR-219 ↑	CaMKIIγ ↓	Kunming mice	CFA and CCI	Prevented and reversed neuropathic pain and spinal neuronal sensitization induced by CFA.	Pan et al. (2014)
	miR-124a ↑	MeCP2 ↓	C57BL/6 mice	Formalin	Decreased inflammatory nociception.	Kynast et al. (2013)
	miR-103 ↑	Cav1.2-LTC ↓	Wistar rats	SNL	Successfully relieve pain.	Favereaux et al. (2011)
	miR-32-5p ↑	Cav3.2 ↓	SD rats	CCI	Reversed mechanical allodynia.	Qi et al. (2022)
	miR-183 ↑	α2δ-1 and α2δ-2	C57BL/6 mice	SNI	Prevented elevation of basal mechanical sensitivity in nociceptors	Peng et al. (2017)

RAP1A, Ras-related protein 1A; DPNP, diabetic peripheral neuropathic pain; CSF1, Colony-stimulating factor 1; SD, Sprague–Dawley; SNL, sciatic nerve injury; IRAK, IL-1R-associated kinase; TRAF6, Tumor necrosis factor receptor-associated factor 6; CCI, chronic constriction nerve injury; HMGB1, high mobility group box 1; CXCR4, Chemokine CXC receptor 4; BAMBI, bone morphogenetic protein and activin membrane-bound inhibitor; TGF-α, Tumor necrosis factor α; CCL2, CC chemokine ligand 2; CCR2, CC chemokine receptor type-2; ZEB1, Zinc finger E-box-binding homeobox 1; TXNIP, Thioredoxin interacting protein; NLRP3, NOD-like receptor protein 3; S1PR1, Sphingosine-1-phosphate receptor 1; RASA1,RAS P21 protein activator 1; KDM3A, lysine-specific demethylase 3A; STAT, signal transducer and activator of transcription; SOCS, Suppressor of cytokine signaling; TRPA1, transient receptor potential cation channel subfamily A member 1; Mtf1, Mitochondrial Transcription Factor 1; OXL, Oxaliplatin; DPN, diabetic peripheral neuropathy; CFA, Complete Freund’s adjuvant; OA, osteoarthritic; XIAP, X-linked inhibitor of apoptosis protein; SNI, sciatic nerve injury; EFNA3, ephrin-A3; SNC, sciatic nerve crush; KLF4, kruppel-like factor 4; ONI, optic nerve injury; RGC, retinal ganglion cell; JAK, Janus kinase gene; STAT, signal transducer and activator of transcription; SCI, spinal cord injury; SPRR1A, small proline-rich repeat protein 1A; PTEN, phosphatase and tensin homolog; ONC, Optic nerve crush; TGFβI, transforming growth factor-beta-induced protein; TIMP3, tissue inhibitor of metalloproteinases 3; EPHA4, erythropoietin-producing human hepatocellular receptor A4; EGFR, epidermal growth factor receptor; mTOR, mechanistic target of rapamycin; GSK3β, glycogen synthase kinase 3β; Ntn1, netrin-1; Dcc, deleted in colorectal cancer; FoxP1, forkhead box protein P1; SIRT1, Sirtuin 1; Nav, voltage-gated sodium channels; DMM, destabilization of the medial meniscus; Kv, voltage-gated potassium channels; TREK-1, TWIK-related K channel 1; MeCP2, Methyl-CpG binding protein 2; Cav1.2-LTC, Cav1.2-comprising L-type calcium channel; CaMKII, calmodulin (CaM)-activated kinase II.

### The regulation of neuroinflammation in neuropathic pain development

MiRNAs have been demonstrated to be involved in almost all known biological processes and many pathophysiological conditions, including neuropathic pain. The activation of thermoreceptors and mechanoreceptors in sensory neurons, which takes place in chronic inflammatory pain conditions, leads to the pathophysiology of neuropathic pain, which includes polyneuropathies, fibromyalgia syndrome, complex regional pain syndrome (CRPS), and postherpetic neuralgia (PHN) (Sayed and Abdellatif, 2011). Numerous miRNAs are becoming more widely recognized as master switches in the pathophysiology condition and as regulators of various neuroinflammation and neuronal gene expression (Figure 2). Differential expression of various miRNAs has been directly reported in the dorsal root ganglion (DRG) after the induction of inflammatory and neuropathic pain (Fang et al., 2022). Therefore, it is necessary to discuss how miRNAs regulate the infiltration of immune cells and neuroinflammation after nerve injury.

#### Immune cell infiltration

It is well known that neuropathic pain is associated with immediate immune cell infiltration following peripheral nerve injury. Recent studies suggest that miRNAs secreted from immune and nonimmune cells exert a pivotal effect on immune regulation (Figure 2). miR-590-3p regulates the infiltration of immune cells into neural tissues in diabetic peripheral neuropathic pain (Wu et al., 2020). Similar to this, after spinal nerve ligation (SNL), there was a decrease in miR-214-3p in the spinal astrocytes of rats, which caused them to become overactive by upregulating CSF1 (Liu L. et al., 2020)*.* Additionally, alterations in the microglial cells’ miRNA profiles imply that these cells have distinct functions depending on the tissue and/or the stage of the pathology.

#### Neuroinflammation

Early research shown that miRNAs can either activate or suppress the immune system, suggesting that they may play a part in the onset and progression of inflammatory and autoimmune illnesses, including neuropathic pain. During the early-to-late phases of the illnesses, there have also been reports of changes in the expression of certain miRNAs (Figure 2). By signalling through the IRAK/TRAF6, TLR4/NF-κB, TXNIP/NLRP3 inflammasome, MAP kinase, and TNF-α and TLR5 signalling pathways, miRNAs mediate their effects in neuropathic pain (Gada et al., 2021).

One of the most crucial receptors for innate immunity, TLR stimulates the generation of pro-inflammatory cytokines, initiates the synthesis of inflammatory mediators that cause fever, pain, and other inflammation, and exerts some regulatory control over the inflammatory response (Tang et al., 2023). Tumour necrosis factor receptor-associated factor 6 (TRAF6), a critical mediator of TLR signalling, NF-κB activation, and proinflammatory cytokine and interferon expression, was upregulated after exposure to TNF-α or IL-1β in cultured astrocytes from mice following SNL. The protein known as IL-1 receptor-associated kinase (IRAK) is involved in the signaling of MyD88. The link between IRAK and TRAF6 encourages additional nuclear factor κ-activated B-cell light chain enhancer (NF-κB) and Janus kinase N-terminal region activation (JAK) (Zarezadeh Mehrabadi et al., 2022). By inhibiting IRAK1 and TRAF6 mRNA 3’UTR sections in TLR signaling pathways and lowering their protein production, targeting miRNA-146a-5p has a detrimental impact on NF-κB activation, the NLRP3 inflammasome signaling pathway, and NP levels (Wang et al., 2018; Hou et al., 2021). miR-381 overexpression alleviated neuropathic pain behaviours in chronic constriction nerve injury (CCI) rats by inhibiting the expression of HMGB1. Moreover, this effect was reversed by miR-381 inhibitors (Xia et al., 2018; Zhan et al., 2018). Similarly, overexpression of miR-362-3p significantly suppressed the elevation of the levels of proinflammatory cytokines by regulating the expression level of BAMBI, which in turn hindered the neuroinflammatory process and NP in CCI mice (Zhang et al., 2022).

Members of the transforming growth factor β (TGF-β) family are secreted cytokines that control a range of biological processes, including as cell division, migration, survival, and differentiation (Nickel et al., 2018). The TGF-family includes transforming growth factor α (TNF-α), which is closely related to NP. A member of the C-C chemokine family, C-C motif chemokine ligand 2 (CCL2) has a strong affinity for C-C chemokine receptor type 2 (CCR2). According to a recent study, overexpression of miR-183 inhibits the TGF-α/CCL2/CCR2 signalling axis, which in turn inhibits the expression of proinflammatory cytokines (IL-6, IL-1β, TNF-α), as well as pain-related markers (TRPV1, Nav1.3, Nav1.7, Nav1.8), in the DRG (Tao et al., 2021). Moreover, zinc finger E-box-binding homeobox 1 (ZEB1) is a transcription factor that is involved in various diseases by inhibiting ZEB1 expression. miR-28-5p, miR-128-3p, miR-136, miR-150, miR-200b and miR-429 can coordinate the progression of neuroinflammation and neuropathic pain by inhibiting ZEB1 expression (Bao et al., 2018; Yan et al., 2018a,b; Shen et al., 2019).

The G-protein-coupled receptor family, which includes the chemokine CXC receptor 4 (CXCR4), controls the development of glia cells and neurons in the central nervous system (Bianchi and Mezzapelle, 2020). The involvement of CXCR4 in various nociceptive stimulus response mechanisms is becoming more and more clear. A multifunctional protein called thioredoxin-interacting protein (TXNIP) is necessary for numerous cellular functions including metabolic processes, growth, division, and cell death (Wondafrash et al., 2020). And inflammation is brought on by the intracellular complex known as the NOD-like receptor family pyrin domain-containing protein 3 (NLRP3) inflammasome, which promotes the development and release of the pro-inflammatory cytokines interleukin-1 (IL-1) and IL-18 (He et al., 2016). They all contribute significantly to the onset of inflammation. MiR-23a controls NP in spinal glial cells by specially targeting CXCR4 and the TXNIP/NLRP3 inflammasome axis. To reduce hyperalgesia, intrathecal injection of miR-23a mimicked spinal CXCR4 downregulation by a lentivirus and blocked TXNIP or NLRP3 overexpression (Pan et al., 2018).

When rats were used as a model for NP, the expression of miR-140 and miR-144 was downregulated in the DRG. Furthermore, by targeting sphingosine-1-phosphate receptor 1 (S1PR1) and RASP21 protein activator 1(RASA1), respectively, intrathecal injection of miR-140 and miR-144 agomir resulted in decreased inflammatory factor secretion and ameliorated hyperalgesia (Zhang et al., 2020; Li et al., 2021). Additionally, miR-216a-5p reduced the neuropathic pain that rats experienced after CCI by targeting KDM3A and deactivating the Wnt/β-catenin signalling pathway (Wang and Li, 2021). Signal transducer and activator of transcription 3 (STAT3)’ 3’UTR is the direct target of miR-93, which prevents the disease from developing in CCI rats (Yan et al., 2017).

NF-κB is a key mediator in the inflammatory process. The M1 and M2 macrophages are activated by the activation of the NF-κB pathway, which causes them to release pro-inflammatory cytokines and speed up the inflammatory response process (Poladian et al., 2023). Meanwhile, MAPK is a mitogen activated kinase that controls a number of physiological and pathological processes, including NP, by phosphorylating serine/threonine and tyrosine (Zhang et al., 2023). However, the balance of Th1-Th2 cells, the control of cytokine signaling negative feedback, and the reduction of Th2-induced inflammation are all regulated by a unique family of proteins known as cytokine signaling inhibitors (SOCS). Several inflammatory and anti-inflammatory cytokines stimulate SOCS1 and SOCS3, which then block cytokine function (Sobah et al., 2021). Many researchers noted that miR-155 or miR-221 inhibition alleviated neuropathic pain and neuroinflammation by enhancing suppressor of cytokine signalling 1 (SOCS1) expression *via* NF-κB and p38-MAPK inhibition (Tan et al., 2015; Xia et al., 2016; Liu Y. et al., 2021). Additionally, inhibition of miR-221 reduced pain and decreased the expression of inflammatory factors (PEG2, BK, IL-6, IL-1β, and TNF-α) by targeting SOCS3 in diabetic peripheral neuropathy (DPN) (Wu et al., 2021). Additionally, spinal cord miR-155 expression was increased in oxaliplatin-induced peripheral neuropathic pain, and the intrathecal injection of a miR-155 inhibitor reduced hyperalgesia in rats, potentially through blocking oxidative stress-TRPA1 pathways (Miao et al., 2019). Complete Freund’s adjuvant (CFA)-induced mechanical allodynia and heat hyperalgesia were reduced by knocking down or blocking miRNA-22, but overexpressing miRNA-22 resulted in pain-like behaviours. In order to activate RNA polymerase II and elevate Mtf1 expression, the enhanced miRNA-22 physically bonded to the Mtf1 promoter. Increased expression of p-ERK1/2, GFAP, and c-Fos in the dorsal horn is proof that the increased Mtf1 expression faciliated spinal central sensitization (Hao et al., 2022).

These results suggest that epigenetic interventions against miRNAs to alleviate neuroinflammation may potentially provide novel therapeutic avenues in treating peripheral nerve injury-induced nociceptive hypersensitivity and neuropathic pain.

### Regulation of nerve regeneration

After peripheral nerve injury, the survival of neurons is an essential prerequisite for neural regeneration and functional recovery. According to earlier research, prolonged pain causes spinal cord and peripheral nerve cell body damage or possibly cell death (Cohen et al., 2021).

Recent studies have identified that injured peripheral neurons can activate the secretion of intrinsic neurotrophic factors that promote neuronal survival and axon regeneration, such as nerve growth factor (NGF), brain-derived neurotrophic factor (BDNF), and neurotrophin-3 (NT-3) (Keefe et al., 2017). In this regard, miRNAs can also play a role in nerve regeneration (Figure 2). Coincidentally, it was discovered that the DRG has aberrant expression of a number of miRNAs that target genes involved in nerve regeneration (Bai et al., 2007). Moreover, it was suggested that, by increasing the expression of the X-linked inhibitor of apoptosis protein (XIAP), downregulation of miR-192-5p can decrease the apoptosis of nerve cells and aid in the regeneration process following sciatic nerve injury (SNI) (Liu X. et al., 2020). Additionally, the overexpression of miR-210 contributed to neuronal survival by preventing apoptosis through targeting ephrin-A3 (EFNA3), thereby promoting the regeneration of axons (Hu et al., 2016). Kruppel-like factor 4 (KLF4), a really well intrinsic inhibitor of axonal outgrowth and regeneration, can be efficiently silenced by miR-135a and miR-135b to stimulate axonal outgrowth and cortical neuron migration (van Battum et al., 2018).

In spinal cord injury (SCI) rats, direct 3D fiber hydrogel scaffold implantation combined with continuous supply of a cocktail of axon miRNAs (miR-132, miR-222, and miR-431) dramatically improved axon regeneration (Zhang et al., 2021). In earlier research, miR-125b overexpression supported axon regeneration after spinal cord injury through controlling the JAK/STAT pathway. Furthermore, through lowering neuronal apoptosis and the inflammatory reaction, miR-125b demonstrated a neuronal protective effect (Dai et al., 2018). *In vivo*, miR-155 deletion enhanced injury-induced expression of SPRR1A, a regeneration-related gene, in neurons and reduced inflammatory signalling in macrophages, thereby enhancing axon regeneration (Gaudet et al., 2016). Mice with an optic nerve compression greatly increased their ability to regenerate their axons *in vivo* by enhancing the levels of miR-19a in their retinal ganglion cells (Mak et al., 2020). Additionally, injury-induced changes in the expression of miR-21 and miR-199a-3p changed the ability of axons to develop by altering both systemic and intra-axonal protein synthesis through control of the PTEN/mTOR pathway (Kar et al., 2021). The PTEN/mTOR pathway is a major factor in determining axonal regeneration. The tumor suppressor phosphatase and tensin homologue (PTEN) is a PIP3 phosphatase, and an inhibitor of mTOR signaling. The serine/threonine protein kinase known as the mechanistic target of rapamycin (mTOR), whose activation improved protein synthesis and mRNA translation (Tang, 2018). They synergistically regulate axon growth. MiRNA-21 controls the expression of TGF-I, TIMP3, and EPHA4 target genes, which is crucial for increasing Schwann cell (SC) proliferation and axon regeneration during the healing of damaged peripheral nerves (Ning et al., 2020). Moreover, endogenous miR-26a in sensory neurons enhanced the regeneration of sensory axons after spinal cord injury (SNI) by promoting the induced activation of Smad1 and inhibiting the expression of glycogen synthase kinase 3β (GSK3β) (Jiang et al., 2015).

In contrast, many miRNAs can also negatively regulate neuronal regeneration, thereby supporting neuropathic progression. By controlling the epidermal growth factor receptor (EGFR) pathway, miR-21, for instance, promotes the hyperactivation of astrocytes and the development of glial scar tissue, preventing the regeneration of axons (Li et al., 2018). Additionally, miR-455-5p inhibition suppressed axonal growth and regeneration and downregulated activation of the GSK3β/Tau protein pathway in murine sensory neurons (Su et al., 2020). miR let-7 and miR-9 hindered axonal regeneration through inhibition of the protein levels of Ntn1 and Dcc (Wang et al., 2019), respectively. Likewise, researchers found that axon regeneration is also driven by miR-9 through regulation of FoxP1 triggered by injury. However, sensory neurons with high endogenous miR-9 levels were unable to regenerate their axons (Jiang et al., 2017). Additionally, a novel mechanism for regulating the capacity for intrinsic axon regeneration is provided by the mutual negative response regulatory loop formed by miR-138 and SIRT1 (Liu et al., 2013).

These findings present a novel idea for the future study of axon regeneration in neuropathic pain. There are still many obstacles to be overcome in order to create therapies that achieve complete regeneration and functional recovery of neurons, even though substantial advances have been made in comprehending the fundamental mechanisms of peripheral nerve regeneration and how these pathways can be effectively utilized to promote regeneration after peripheral nerve injury (PNI).

### Regulation of neuronal ion channels

After the nerve fiber is injured, the structure and function of ion channels in the nerve endings and DRG of the spinal cord may change, leading to the ectopic discharge of neurons, and neuropathic pain (Jiang et al., 2022). Thus, ion channels play a key role in neuronal excitability and may be targets of miRNAs under pain conditions (Figure 2). Notably, voltage-gated channels involved in the pain pathway have become the main targets of neuropathic pain treatment interventions.

#### Voltage-gated sodium channels

Voltage-gated sodium channel Nav1.3, an isoform that is sensitive to tetrodotoxin and is encoded by SCN3A, can produce sodium ion currents with quick repriming dynamics. These currents can promote repetitive firing patterns and ectopic discharge in damaged neurons, which can enhance neuronal hyperexcitability and are strongly linked to neuropathic pain (Lindia et al., 2005). Similar sodium-ion channels also have Nav1.6, encoded by SCN8A, and Nav 1.7, encoded by SCN9A (Bennett et al., 2019).

MiR-96 administered intrathecally inhibited the expression of Nav1.3 brought on by CCI. Further investigation indicated that miR-96 decreased the *in vitro* expression of Nav1.3 mRNA in embryonic DRG neurons (Chen et al., 2014). By controlling SCN3A, miR-384-5p inhibits the emergence of neuropathic pain (Ye et al., 2020). By controlling Nav1.7 in rats, miR-182 reduced the neuropathic pain brought on by SNI (Cai et al., 2018). Surprisingly, overexpression of miR-30b lowered the expression of Nav1.3, Nav1.6, and Nav1.7 both in DRG neurons and the spinal cord, which greatly reduced neuropathic pain brought on by SNL or oxaliplatin (Shao et al., 2016; Su et al., 2017; Li L. et al., 2019). Consistently, miR-183 overexpression attenuated osteoarthritic pain by inhibiting the production of Nav1.3, Nav1.7, and Nav1.8 (Tao et al., 2021).

miR-7a overexpression in primary sensory neurons of injured DRGs suppressed the increase in the β2 subunit of the voltage-gated sodium channel and normalized the long-lasting hyperexcitability of nociceptive neurons (Sakai et al., 2013).

#### Voltage-gated potassium channels

Voltage-gated potassium channels play a crucial role in controlling the excitability of neurons by altering the production of action potentials, the pace at which neurons fire, or the release of neurotransmitters (Kim and Nimigean, 2016). miR-17-92, a miRNA cluster that includes six different members, downregulated the expression of potassium channels and reduced outward potassium currents, especially type A currents, resulting in the generation of mechanical allodynia (Sakai et al., 2017). Additionally, blocking miR-137 reduced mechanical allodynia and thermal hyperalgesia, recovered aberrant Kv currents and the overactivity of DRG neurons, and restored the expression of the potassium channel Kv1.2 (Zhang et al., 2021). Additionally, miR-183-5p contributed to the control of CCI-induced NP by suppressing TREK-1 expression (a Kv channel) (Shi et al., 2018).

#### Calcium channels

In addition to voltage-gated sodium channels and potassium channels, calcium channels also play an indispensable role in the process of pain sensitization after nerve injury due to their involvement in neurotransmitter release and the regulation of neuronal excitability and intracellular changes, including gene induction (Rahman and Dickenson, 2013). MiR-219 and miR-124a, which negatively influenced the expression of spinal CaMKIIγand the proinflammatory marker MeCP2, were dramatically downregulated in murine spinal neurons following the development of inflammatory pain by either CFA or formalin injection (Kynast et al., 2013; Pan et al., 2014). Additionally, miR-103 expression was shown to be downregulated in spinal neurons of SNL rats, which appeared to simultaneously control the translational levels of the three components that make up the Cav1.2-comprising L-type calcium channel (Cav1.2-LTC), a calcium ion channel associated with pain sensitization (Favereaux et al., 2011). Additionally, by targeting Cav3.2 channels, histone methylation-mediated miR-32-5p decreased expression in trigeminal ganglion (TG) neurons controls trigeminal NP (Qi et al., 2022). By controlling the auxiliary voltage-gated calcium channel subunits α2δ-1 and α2δ-2, the miR-183 cluster in mice regulated more than 80% of NP-regulated genes and attributed to scaling basal mechanical sensitivity and mechanical allodynia (Peng et al., 2017).

These findings suggest that miRNA-mediated channel dysfunction is a significant contributor to the pathogenesis of nerve injury-induced NP, highlight the significance of abnormal afferent input in the persistence of neuropathic pain and the promise of targeted chemogenetic silencing as a potential neuropathic pain therapy.

### Role of exosomal miRNAs in neuropathic pain

Exosomes (Exos), a class of nanosized EVs with sizes from 40 to 200 nm, are released from all cell types and participate in paracrine interactions between various cells, including neurons, glial cells, mesenchymal stem cells, endothelial cells, and leukocytes (Mathivanan et al., 2010). As another type of secreted factor, these biological nanocarriers, which are rich in a variety of genetic materials, including miRNAs, long noncoding RNA, proteins, and lipids, can be easily distributed in biofluids and modulate biochemical responses and cell viability during physiological and pathological conditions in neurodegenerative or inflammatory diseases (Cata et al., 2022). Blood, saliva, breast milk, urine, and other bodily fluids all contain significant amounts of exosomal miRNAs (Zhang et al., 2015; Ding et al., 2019).

**Figure 3:**
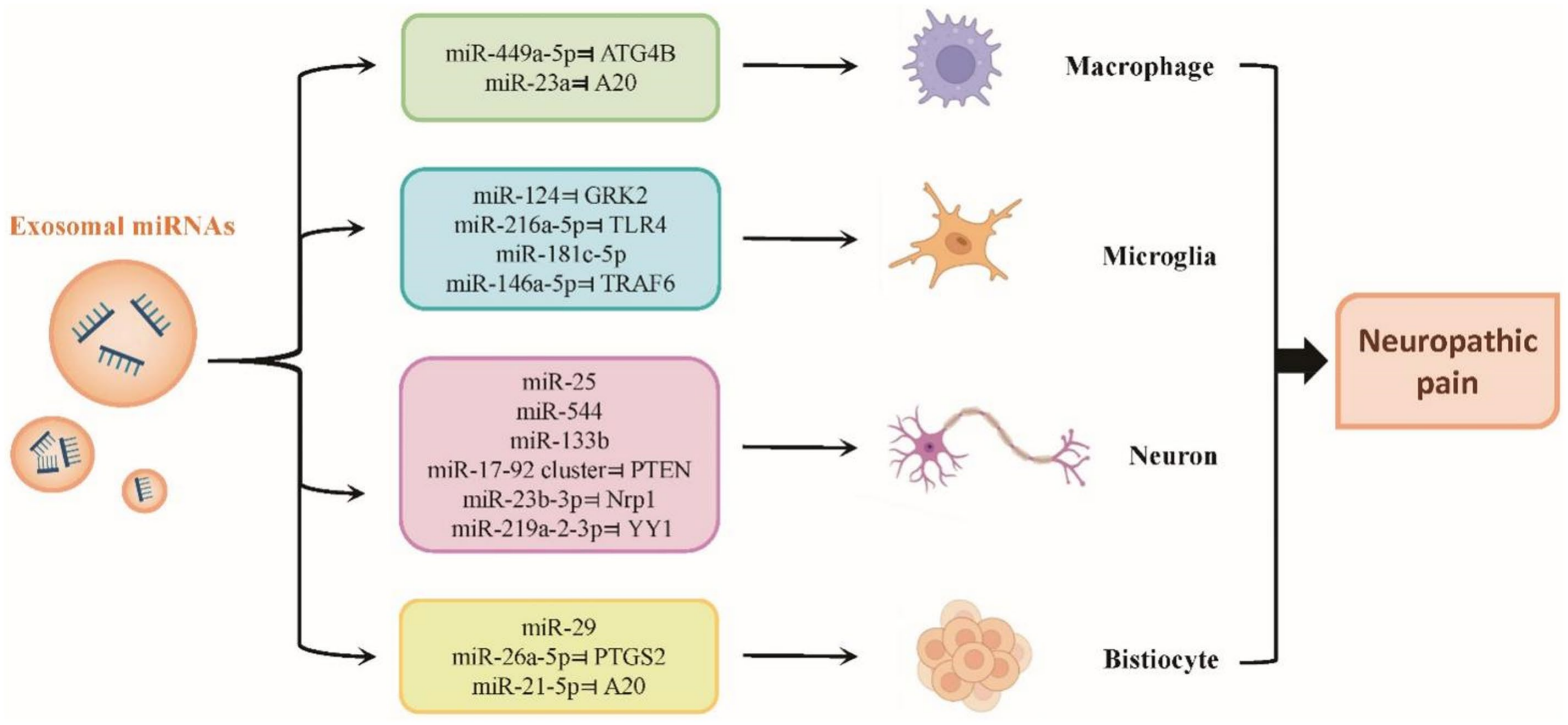
Roles of exosomal miRNAs in neuropathic pain. Exosomal miRNAs can be transported to different sites after autocrine production, acting on macrophages, microglia, neurons or other tissue cells, and regulate the process of neuropathic pain by participating in the secretion of inflammatory factors, and oxidative stress, and regulating neural remodelling or nerve regeneration.

Pain is frequently caused by inflammation. A variety of cytokines, chemokines, and additional elements contribute to the emergence of acute inflammatory pain. Chronic inflammation can start the processes that lead to cerebral and peripheral sensitization (Jiang et al., 2020). Exosomal miRNAs can be transported to different sites after autocrine production, acting on macrophages, microglia, neurons or other tissue cells, and regulate the process of neuropathic pain by participating in the secretion of inflammatory factors, and oxidative stress, and regulating neural remodelling or nerve regeneration (Figure 3). Exosomes have the ability to regulate the release from cells of nociceptive mediators, which are involved in neuroinflammation and are recognized to sensitize sensory terminals (Groot and Lee, 2020). For instance, immunological cells such as T lymphocytes and antigen-presenting dendritic cells (DCs) can release and absorb exosomal miRNAs, indicating that exosomal transfer of miRNAs may constitute a novel method of intercellular communication (Torralba et al., 2018). As a result, it is believed that exosomal miRNA transmission is significant for a number of systems and processes, such as the immune reaction and neuron–glia communication (Kalani et al., 2014).

Interestingly, these exosomal miRNAs can have both proinflammatory and anti-inflammatory effects. As mentioned above, some exosomal miRNAs can release cytokines or other proinflammatory mediators that directly act on target organs (Zhang et al., 2014; Console et al., 2019). For example, exosomes from chondrocytes, neutrophils, and synovial fibroblasts encouraged macrophages to produce IL-1 and metalloproteinases. Furthermore, the regulation of the synthesis of these inflammatory chemicals has been linked to the transfer of miR-206 and miR-449a-5p in the cargo of exosomes (Liu et al., 2018; Ni et al., 2019). MiR-449a-5p in particular mediates ATG4B inhibition, which in turn controls macrophage autophagy, encourages inflammosome activation, and exacerbates the inflammatory response.

Evidence suggests that miR-21 was increased in serum exosomes from neuropathic mice. Further research revealed that miR-21-5p-containing macrophage-derived exosomes promoted pyroptosis *via* A20, encouraging a proinflammatory phenotype and exacerbating podocyte damage in diabetic nephropathy mice (Ding et al., 2021). Notably, the DRG’s hyperalgesia and macrophage recruitment were reduced by either intrathecal miR-21-5p antagomir addition or miR-21 conditional deletion in sensory neurons. A20 is an inhibitor of the NF-κB signaling pathway. Similar research showed that after nerve damage, DRG sensory neurons released EVs that were miR-23a-enriched and were then ingested by macrophages to improve M1 polarization *in vitro*. Additionally, by blocking A20 to promote NF-κB signaling, an EV-miR-23a antagomir delivered intrathecally reduced M1 macrophages and improved neuropathic hyperalgesia (Zhang et al., 2021).

Conversely, other exosomal miRNAs exert anti-inflammatory and analgesic effects in chronic pain models *via* the transfer of therapeutic factors to injured neurons in the central nervous system (CNS) and peripheral nervous system (PNS) (Ren J. et al., 2019). The promotion of inflammation is thought to be the leading cause of pain. Exosomal miRNAs can also suppress the production of proinflammatory cytokines such as IL-1β, IL-6, TNF-α, and PGE2 in injured areas and stimulate the release of IL-10, leading to antinociceptive effects (Cata et al., 2022). These miRNAs have the ability to modify nociception, and intrathecal administration of miR-124, miR-103, miR-23b, miR-25, and miR-544, for example, reduced inflammatory and neuropathic pain by modifying intracellular neuronal, astrocytic, and microglial activities (Favereaux et al., 2011; Willemen et al., 2012; Wang et al., 2018; Zhao et al., 2019; Li et al., 2020). MiR-124 inhibits GRK2 expression, thereby regulating the M1/M2 phenotypic balance of the spinal cord.

Similar to the effect of some neurotrophic factors, including GDNF, IGF-1, BDNF, NGF, and FGF-1, exosomal miRNAs can enhance axonal growth and neuronal viability and intensify therapeutic effects (Lv et al., 2021). To the best of our knowledge, the targeted and modular EV loading (TAMEL) method has not been implemented in experimental pain studies. Despite the fact that many studies have tried to provide new and more effective mechanistic insights into the function of exosomal miRNAs in NP, the cellular and molecular functions of exosomal miRNAs and their downstream targets remain to be elucidated.

## Emerging role of exosomal miRNAs in neuropathic pain management

Standard analgesics, such as acetaminophen, nonsteroidal anti-inflammatory drugs, local anesthetics, and, to a lesser extent, opioids, may be helpful in controlling acute pain. Unfortunately, due to ineffectiveness or undesirable side effects, there are few clinically useful analgesics for the treatment of neuropathic pain (Amaechi et al., 2021). Therefore, present research investigations should give top priority to the identification and development of mechanism-based therapies for the amelioration of neuropathic pain.

Exosomes have the capacity to control NP and can be secreted by a variety of cell types, including stem cells, according to laboratory research (Ren J. et al., 2019). Further evidence suggests that stem cell-derived exosomes can largely mimic the functional effects of parental stem cells and have been identified as key players in the stem cell repair of damaged tissues (Keshtkar et al., 2018). Stem cell-derived exosomes can not only play a role in nerve repair but also avoid the risk of immunosuppression, genetic modification and malignant transformation caused by stem cell transplantation due to their paracrine effect, providing a new therapeutic strategy and research target for neuropathic pain (Liu W. Z. et al., 2021). It is believed that stem cell-derived exosomes can transfer neurotrophic factors, such as GDNF, IGF-1, BDNF, NGF, and FGF-1, to injured neurons. Additionally, intrathecal infusion of mesenchymal stem cell exosomes reduces neuropathic pain in spinal cord injured rats by causing microglia to become polarized from M1 to M2 and preventing the release of inflammatory cytokines such TNF-α, IL-1, IL-6, and NF-κB (Harrell et al., 2019; Arabpour et al., 2021; Liu W. Z. et al., 2021). Among them, the regulatory effect of the miR-216a-5p/TLR4 axis on microglial polarization has been demonstrated (Liu W. et al., 2020).

**Figure 4:**
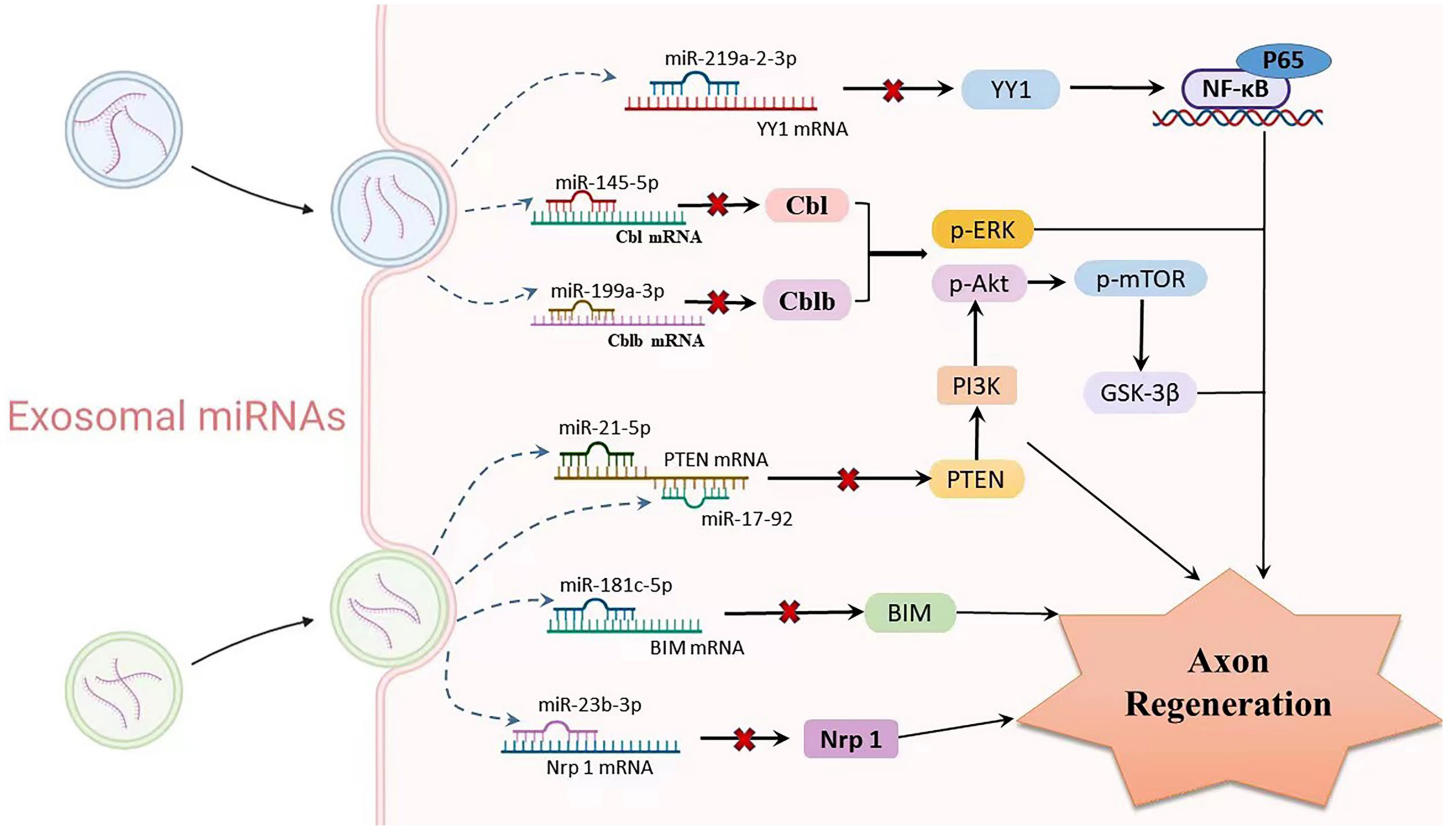
Relationship between exosomal miRNAs and axon regeneration. As one of the important cargos of exosomes, miRNAs are involved in mediating the process of axon regeneration. Exosomes produced by various stem cells enter neurons or glial cells through paracrine routes, where multiple miRNAs are released. Upregulation of exo-miR-219a-2-3p inhibits YY1 expression, thereby suppressing the NF-κB-p65 pathway and exerting neuroprotective effects. miR-199a-3p/145-5p suppresses Cblb and Cbl expression and causes upregulation of p-Erk and p-Akt, subsequently promoting neurite outgrowth. The miR-17-92 cluster and miR-21-5p carrying exosomes downregulate PTEN levels and subsequently activate the PI3K/Akt/mTOR signalling pathway, thereby increasing neuronal dendritic plasticity. MiR-181c-5p negatively regulates BIM mRNA, inhibits BIM expression level, reduces neuronal apoptosis, and promotes axon regeneration. Targeted inhibition of Nrf1 mRNA levels by miR-23b-39 subsequently promotes axonal regeneration.

Mesenchymal stem cells (MSCs) are one of the most promising stem cell types for the treatment of various ischaemic diseases and tissue damage due to their multidirectional differentiation potential and extensive immune regulatory functions (Uccelli et al., 2008). A recent laboratory study indicated that MSCs can migrate to the injured nerve tissue and stimulate the regeneration of injured neurons (Norte-Muñoz et al., 2021). MSC-derived exosomes regulate neurite growth by controlling the number and total length of neurites through the transfer of miR-133b to nerve cells (Xin et al., 2012, 2013; Ren Z. W. et al., 2019). Exosomes enriched in miR-17-92 clusters may increase neuroplasticity and functional recovery by targeting PTEN to activate the PI3K/Akt/mTOR/GSK-3β signalling pathway (Xin et al., 2017). Similarly, SC-derived small exosomes containing miR-21-5p negatively regulate PTEN to improve sensory neuron growth and survival (Cong et al., 2021). In order to increase the capacity for neurite outgrowth *in vitro* and nerve regeneration *in vivo*, SCs-extracellular vehicles (EVs) transported miR-23b-3p from mechanically activated SCs to neurons and decreased neuronal neuropilin 1(Nrp1) expression (Xia et al., 2020). Umbilical cord mesenchymal stem cell-derived exosomes boost axon regrowth and spinal cord functional improvement *via* miR-199a-3p/145-5p targeting of Cblb/Cbl-mediated NGF/TrkA signaling in rats (Wang et al., 2021). Following spinal cord damage, exosomes released from neural stem cells exposed to IGF-1 decreased apoptosis and promoted nerve regeneration, at least in part through a miR-219a-2-3p/YY1 mechanism (Ma et al., 2019). Similarly, miR-181c-5p negatively regulates Bcl-2-interacting cell death mediators (BIM), which can effectively inhibit neuronal apoptosis and regulate the cell vitality of cortical neurons to promote axon regeneration (Zhang et al., 2019; Figure 4).

A recent study suggested that miR-29-enriched exosomes originate from stem cells and alleviate proinflammatory responses in an osteoarthritis in a rat model (Le et al., 2016). Similarly, miR-199-3p overexpression attenuated TRX-induced PHN by targeting MECP2 in mice (Wang et al., 2022). ExomiR-181c-5p was incorporated by microglial cells and prevented inflammatory substances from being released. In addition, intrathecal exomiR-181c-5p treatment reduced neuroinflammatory symptoms and neuropathic pain in CCI rats (Zhang et al., 2022). In order to reduce neuroinflammation, human umbilical cord MSC-derived exosomes upregulated the expression of proteins associated with autophagy (LC3-II and beclin1) and blocked the activation of NLRP3 inflammasomes through miR-146a-5p/TRAF6 in the spinal cord dorsal horn (Hua et al., 2022). Furthermore, cyclooxygenase-2 (PTGS2) was downregulated in rat synovial fibroblasts after xenogenic injection of human MSC exosomes enriched with miR-26a-5p to lessen pathogenic alterations (Jin et al., 2020).

MSC-derived exosomes can exert analgesic effects in chronic pain models *via* the transfer of certain miRNAs in the CNS and PNS (Hmadcha et al., 2020). Exosomal miRNAs can be important biomarkers, and compared with free-floating miRNAs, they have the following advantages: (Scholz et al., 2019) exosomes contain a range of miRNAs, making them reliable carriers for the study of miRNAs; (Yi et al., 2021) the bilayer membrane shape of exosomes can improve miRNA stability, susceptibility to miRNA amplification, and the likelihood of unfavorable outcomes; and (Huh et al., 2017) exosomes can cross the blood–brain or blood-spinal cord barriers (Ding et al., 2019). Despite the potential advantages of MSC-derived exosomes, the low therapeutic effect due to poor survival of transplanted cells in damaged tissues is still the largest obstacle in stem cell therapy.

These data suggest that stem cell-derived exosomal miRNAs can manage pain by reducing proinflammatory cytokines and promoting neuronal regeneration and differentiation, which presents a novel therapeutic strategy for the treatment of nerve injury.

## Perspectives on this review

In conclusion, neuropathic pain poses a substantial threat to patients’ lives, health, and quality of life, and the absence of safe and effective treatment options continues to be a significant therapeutic challenge. In recent years, the in-depth study of miRNA in the development of neuropathic pain has provided great opportunities for its clinical transformation, especially its outstanding contribution in neuroinflammation, nerve regeneration and other aspects has brought another bright prospect for the clinical treatment of neuropathic pain. Even though it has been demonstrated that miRNA is a viable candidate for NP therapy, obstacles such its bioactivity, stability, safety, and tissue specificity still need to be overcome. On the journey to the target, miRNA’s transmembrane efficiency and enzymatic reaction are additional factors that must be taken into account. The study of delivery molecules, including exosomes, liposomes, viral vectors, as well as miRNA mimics and inhibitors, is therefore currently given significant emphasis. Last but not least, patient safety has to be the top priority in clinical application, making it difficult for us to effectively assess the immunological response that exogenous miRNA treatment may trigger. Further preclinical research and clinical trials are crucial measures to support clinical transformation because current research is still confined to cell and animal investigations. With further research on the mechanism of action of miRNAs and the use of the latest miRNA gene chips and other high-throughput technologies, miRNAs may become a new biological marker for disease diagnosis and will provide new targets and methods for the pathogenesis and intervention strategies of neuropathic pain in the future.

## Author contributions

Y-YZ wrote the manuscript and made illustrations. Z-JW, L-JZ, and T-XN provided advice for the manuscript. BL and JL provided the supervision and comments on the manuscript. All authors have read and approved the final manuscript.

## Funding

This work was supported by grants from the Tianjin Natural Science Foundation Project (grant no. 20JCYBJC00370), Tianjin, China, and the Tianjin Key Medical Discipline (Specialty) Construction Project (grant no. TJYXZDXK-017A).

## Conflict of interest

The authors declare that the research was conducted in the absence of any commercial or financial relationships that could be construed as a potential conflict of interest.

## Publisher’s note

All claims expressed in this article are solely those of the authors and do not necessarily represent those of their affiliated organizations, or those of the publisher, the editors and the reviewers. Any product that may be evaluated in this article, or claim that may be made by its manufacturer, is not guaranteed or endorsed by the publisher.

## Figures and Tables

**Table:** 

miRISC	miRNA-induced silencing complex
DRG	Dorsal root ganglion
SNL	Spinal nerve ligation model
CSF1	Colony-stimulating factor 1
IRAK	IL-1R-associated kinase
TRAF6	Tumour necrosis factor receptor-associated factor 6
TLR	Toll-like receptors
NF-κB	Nuclear factor κB
TXIP	Thioredoxin-interacting protein
NLRP3	NOD-like receptor protein 3
TNF-α	Tumour necrosis factor α
IL-1β	Interleukin-1β
3’UTR	3′ untranslated region
CCI	Chronic constriction nerve injury
HMGB1	High mobility group box 1
BAMBI	Bone morphogenetic protein and activin membrane-bound inhibitor
NP	Neuropathic pain
TRPV1	Transient receptor potential (TRP) vanilloid 1
TGF-α	Transforming growth factor-α
CCL2	CC chemokine ligand 2
CCR2	CC chemokine receptor type-2
ZEB1	Zinc finger E-box-binding homeobox 1
CXCR4	Chemokine CXC receptor 4
TXNIP	Thioredoxin interacting protein
S1PR1	Sphingosine-1-phosphate receptor 1
RASA1	RAS P21 protein activator 1
KDM3A	Lysine-specific demethylase 3A
STAT3	Activator of transcription 3
SOCS1	Suppressor of cytokine signalling 1
MAPK	Mitogen-activated protein kinase
p38	p38 mitogen-active protein kinase
PEG2	Prostaglandin E2
SOCS3	Suppressor of cytokine signalling 3
DPN	Diabetic peripheral neuropathy
CFA	Complete Freund’s adjuvant
Mtf1	Mitochondrial Transcription Factor 1
p-ERK1/2	Phosphorylation of ERK1/2
GFAP	Glial fibrillary acidic protein
NGF	Nerve growth factor
BDNF	Brain-derived neurotrophic factor
NT-3	Neurotrophin-3
FGF	Fibroblast growth factor
XIAP	X-linked inhibitor of apoptosis protein
EFNA3	Ephrin-A3
KLF4	Kruppel-like factor 4
SCI	Spinal cord injury
JAK	Janus kinase gene
STAT	Signal transducer and activator of transcription
SPRR1A	Small proline-rich repeat protein 1A
PTEN	Phosphatase and tensin homologue
mTOR	Mechanistic target of rapamycin
SC	Schwann cells
GFβI	Transforming growth factor-beta-induced protein
TIMP3	Tissue inhibitor of metalloproteinases 3
EPHA4	Erythropoietin-producing human hepatocellular receptor A4
GSK3β	Glycogen synthase kinase 3β
EGFR	Epidermal growth factor receptor
Ntn1	Netrin-1
Dcc	Deleted in colorectal cancer
FoxP1	Forkhead box protein P1
SIRT1	Sirtuin 1
Nav	Voltage-gated sodium channels
SNL	Spinal nerve ligation model
Kv	Voltage-gated potassium
TREK-1	TWIK-related K channel 1
MeCP2	Methyl-CpG binding protein 2
Cav1.2-LTC	Cav1.2-comprising L-type calcium channel
CaMKII	Calmodulin (CaM)-activated kinase II
TG	Trigeminal ganglion
EVs	Extracellular vesicles
DCs	Dendritic cells
CNS	Central nervous system
PNS	Peripheral nervous system
MS	Mechanical stimulation
Nrp	Neuropilin
PHN	Postherpetic neuralgia
TRX	Thioredoxin
MECP2	Methyl-CpG-binding protein 2
IGF-1	Insulin-like growth factor I
MSCs	Mesenchymal stem cells
PTGS2	Prostaglandin-endoperoxide synthase 2.
